# Requirements for evidence-based management competency in healthcare: a scoping review

**DOI:** 10.3389/fpubh.2025.1490454

**Published:** 2025-01-28

**Authors:** Elyas Sanaeifar, Elaheh Houshmand, Javad Moghri, Marjan Vejdani, Seyed Saeed Tabatabaee

**Affiliations:** ^1^Student Research Committee, Mashhad University of Medical Sciences, Mashhad, Iran; ^2^Department of Management Sciences and Health Economics, School of Health, Mashhad University of Medical Sciences, Mashhad, Iran; ^3^Social Determinants of Health Research Center, Mashhad University of Medical Sciences, Mashhad, Iran; ^4^Iranian Research Center on Healthy Aging, Sabzevar University of Medical Sciences, Sabzevar, Iran

**Keywords:** requirements, evidence-based, management competency, Iran, scope review

## Abstract

**Background:**

Identifying the essential skills and qualifications for evidence-based managers in healthcare is crucial for decision-makers who aim to select competent managers and design effective training programs. This study reviews the requirements, capabilities, and skills necessary for evidence-based managers in the healthcare sector to accurately utilize and implement evidence.

**Method:**

This scoping review was conducted following the PRISMA-SCR (Preferred Reporting Items for Systematic Reviews and Meta-Analyses Extension for Scoping Reviews) guidelines. A thorough literature search was carried out using relevant keywords across the Web of Science, PubMed, and Scopus databases, with no time restrictions. The selection of articles adhered to predefined inclusion criteria. After eliminating duplicates and reviewing titles, abstracts, and full texts, six articles were included in the final analysis.

**Results:**

The study identified several competencies required for effective evidence-based management in healthcare, including professional and technical knowledge, leadership, personal traits and attitudes, communication skills, information management, self-management, critical thinking, research skills, and the ability to apply evidence effectively.

**Discussion:**

Given the ongoing transformations in healthcare, including the emergence of new technologies and the generation of extensive data, evidence-based managers must continually enhance their skills to access and evaluate evidence. They should also work on improving both their interpersonal and intrapersonal skills. The authors advocate for further applied research to deepen our understanding of the competencies required for evidence-based managers within the healthcare and treatment contexts.

## Introduction

In today’s highly dynamic and unpredictable business landscape, leveraging data is crucial for gaining a competitive edge (1). Despite this, many organizational decisions continue to be based solely on experience, without taking advantage of other available data sources, and often despite evidence to the contrary (2). This suggests that while business environments are rapidly transforming, management practices are not keeping pace with the demands of a data-driven world (3).

The approach of evidence-based management (EBM) involves utilizing the most reliable and informed evidence available to make clear and wise decisions in the field of management. To implement this approach, managers must collaborate with stakeholders to collect and analyze evidence for their decision-making (4). Understanding the competencies and characteristics of managers who utilize EBM can help organizations cultivate the necessary capabilities in their management teams (3). By adopting EBM, healthcare organizations can navigate complex challenges and achieve better results through informed decision-making based on the best available evidence (5).

The evidence-based process consists of five steps: 1. problem identification and assignment; 2. compilation of literature and internal evidence; 3. Cross-synthesis of evidence and reformulation of the problem. 4. engage stakeholders and develop evidence-based alternatives; and 5. commit to evidence-based solutions and implementation (6). It seems that for simple issues with predictable results, applying these steps is simple, but when it comes to strategic decisions and long and ambiguous changes, the complexity of using EBM should increase. Using EBM for strategic decision-making can be complex and challenging due to several factors, including resistance to change, the quality and relevance of evidence, and the integration of evidence with individual expertise. One important barrier is the inherent resistance to changing decision-making practices. Managers may prefer traditional methods based on intuition or past experiences rather than adopting a systematic approach that relies on empirical data. This reluctance can stem from a lack of understanding of the benefits of EBM or a fear of the unknown, which requires effective communication and training to foster adoption. The effectiveness of EBM depends heavily on the availability and quality of data. Organizations often struggle to access high-quality, relevant evidence. For example, while an organization may collect customer feedback, this data may be biased and lead to incorrect decisions. Another challenge is the need to integrate evidence with individual expertise and contextual factors. Decision-makers must balance empirical data with professional judgment, customer preferences, and the specific circumstances of their organization. This integration can be complex. For example, a manager may excel at developing services/products but lack the skills to effectively interpret market information, leading to potential inconsistencies in strategic decision-making (7). Here the role of expertise and art of the decision-maker becomes more important, that is, the evidence-based decision-making manager must be a reliable person and a skilled artist who can convince the stakeholders of his judgment with or without explicit presentation of evidence (6).

The decisions made by managers hold significant weight in an organization, particularly in the healthcare sector where people’s well-being is at stake (5). Despite the multitude of challenges faced by the healthcare industry today, including heightened medical needs and shrinking budgets, the role of healthcare managers and the tough decisions they must make continue to grow in complexity.

Hospital managers approach cost, quality, and efficiency as interconnected factors with common underlying elements, in contrast to many health service researchers who tend to focus solely on quality. They prioritize organizational considerations and recognize that improvements in quality can also lead to cost reductions by decreasing redundancies in the care process and promoting standardization across the organization (8).

Achieving high-quality care and lower costs in healthcare requires healthcare organizations to adopt evidence-based practices, where the relationship between management performance and positive outcomes is rigorously monitored and validated by studies. However, many managers find healthcare management research inaccessible and complain about the lack of focus on their needs (9). The proliferation of management books and journals has only made it harder to separate empirical EBM recommendations from unsupported versions (10). To address this, decision-makers need to identify the skill prerequisites of evidence-based managers in healthcare and plan for their training. This study aimed to review the requirements, abilities, and skills of evidence-based managers in health and treatment to ensure the correct implementation of evidence-based practices.

## Method

This study is a scoping review conducted using the methodological framework of Arksey and O’Malley (11) and the PRISMAScR checklist (12).

Based on this 5-step framework, comprehensive coverage of a topic should be provided and the goal is to identify all relevant literature regardless of study design. This study includes the following steps:

Step 1: Identification of research questions.

To identify the main question of the research, consultation, and exchange of opinions with the research team were used. The research questions were designed to include key requirements, capabilities, and attributes of EBM competency. In other words, the questions were selected according to the research objectives.

Research question:

What are the required competencies and abilities of evidence-based managers in the field of health?

Step 2: Identify relevant studies.

### Search strategy and timeframe

The principal investigator and an expert with a background in review studies (an experienced health services manager) helped develop a keyword search protocol. These two researchers independently conducted extensive and comprehensive searches in electronic databases Web of Science, PubMed, and Scopus, regardless of the period until October 7, 2023, to identify related studies. The following keyword combination is used for searching. Additionally, search terms were customized for each database individually. In addition to this, a search in the references of different studies and a manual search was also done to add to the considered studies.

“Evidence-based management,” “Evidence-Based Health Care Management,” “health service manager,” “health service management,” “management competency,” “hospitals, health care organization”.

The inclusion criteria were:

1. published quantitative, qualitative, mixed, and review studies, original texts including theses, articles, and reports, 2. studies published in English, and 3. studies whose full text was used for data extraction in was available.

The results were entered in reference management software (EndNote 21) and duplicates were removed. Two team members reviewed and verified the search results. All search procedures and results were documented.

Step 3: Study selection.

**Figure 1:**
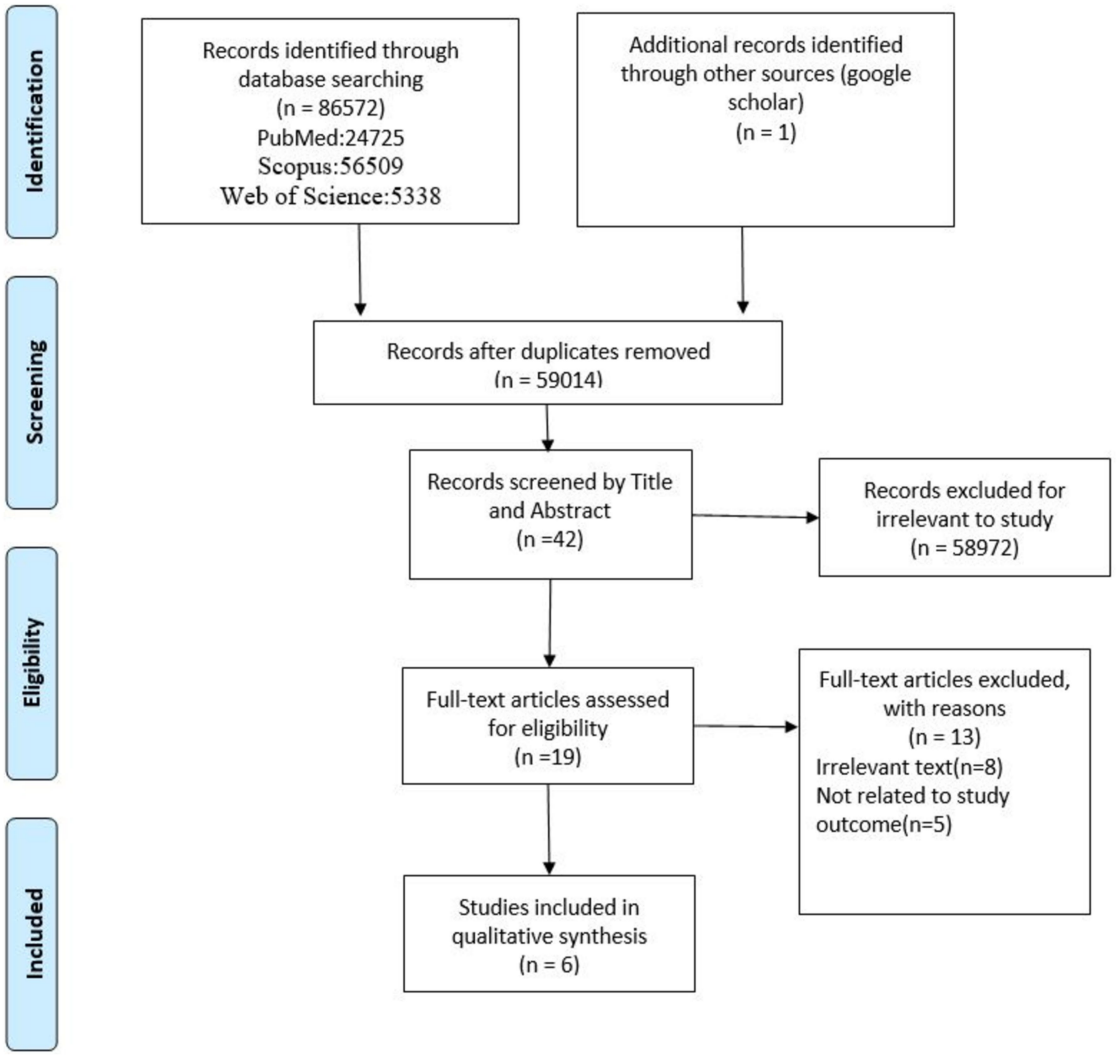
Flowchart of study retrieval and selection process (adapted from PRISMA).

After implementing the search strategy, the first stage of the selection process was carried out. The titles of all studies were reviewed and screened independently by two researchers based on the inclusion criteria. The titles and abstracts of all studies were carefully examined and the titles and abstracts that were most related to the purpose of the study were selected to enter the next stage. A third party resolved disagreements about the authority of documents (3 cases). To assess how the screening process was progressing, a regular discussion was held between members of the research team. These meetings were held at regular intervals to review the study selection process. Irrelevant studies were excluded and the full text of the remaining studies was reviewed. After checking the full text of the studies and matching them, the final studies were selected to be used in the present study. The full text of these studies was independently reviewed by two individuals to confirm their relevance (Figure 1).

Data extracted from each study included the following: title, author (s), publication date (year), study location, study type, document type, and key findings.

Step 4: Collect, summarize, and report the results.

This stage includes collecting, summarizing, and reporting the results. To create and develop a framework for summarizing and synthesizing data and summarizing results, researchers should prioritize certain aspects of the literature (11). This study used the thematic analysis approach to collect and summarize the findings. First, one researcher (E.S.) read all records, annotated them, and identified thematic categories. The same researcher re-read and finalized all records listed in each subject category. To establish reliability, a second researcher (S.T.) verified the analysis for the listed records.

## Results

### General characteristics of final studies

The data collected from the databases are as follows: A total of 5,338 records from Web of Science, 24,735 records from PubMed, and 56,509 records from Scopus. A total of 86,572 original articles and gray texts were found. An overall total of 27,558 duplicate records were identified and removed. By examining the titles of the texts, it was found that 58,972 cases did not meet the inclusion criteria and thus were excluded. The remaining 42 cases were evaluated for their titles and abstracts. A total of 8 records were excluded because they did not align with the research purpose, and 19 full-text articles were retrieved and assessed. Also, the full text of 5 of them was not found. Finally, 6 articles were selected for comprehensive analysis. The oldest study was from 1999 and the most recent study was from 2020. Among the selected articles, 5 studies were original articles (3, 13–16) and one was a review study (17). The studies conducted in Australia were two in number (14, 17), two studies in England (12, 14), one study in America (15) and one study in Lebanon (3). Table 1 shows the specifications of the articles included in the study. Half of the selected studies were from before 2010 and the other half was from after this date. Except for the review study, in 4 articles, the participants were healthcare managers, and in the other study, the participants were researched in the management course. In half of the studies, interviews were used to obtain information.

**Table 1:** General characteristics of the studies included in this research.

Study	Research location	Document	Type of study	Participants
Daouk-Öyry et al. (3)	Lebanon	Original article	Qualitative	Executive managers
Newman et al. (15)	UK	Original article	Qualitative	Chief executives, directors of nursing and quality assurance, business managers, and medical directors
White et al. (16)	US	Original Article	Quantitative	Managers participating in the health administration program
Martins and Isouard (17)	Australia	Review	Review	-
Cowling et al.(13)	UK	Original article	Mix method	Doctors, nurses, midwives, senior managers
Liang et al. (14)	Australia	Original article	Cross-sectional	Middle-level managers

### Extracted topics

After carrying out the research steps, the skills of evidence-based managers extracted from the studies were: professional and technical knowledge (3, 13–17), leadership (3, 14–17), personal characteristics and attitude (3, 13, 14, 16, 17), communication (3, 13–17), information management (13, 14, 17), self-management (3, 13, 17), way of thinking (3, 14, 16), and research and use of evidence (3, 13, 16, 17) (Table 2).

**Table 2:** Requirements, abilities, and skills of evidence-based managers.

#	Item	
1	Professional and technical knowledge (3, 13–17)	General management, financial management and digital skills, national and international standards, process management and quality assurance (3), writing evidence-based protocols and audit skills to monitor the effectiveness of the new model (17), solving job problems, designing and Project management, time management and computing skills (15), identification of risks and problems, identification of internal and external units of the organization, identification of alternatives and processes, identification of the conditions and scale of operations, evaluation of the impact of nurses, definition of the scope of professional standards Financial capabilities, writing clinical protocols, applying processes to increase sustainability in human resources, measuring performance/output and matching evaluation/achievement, organizing effective protocols, organizational design to consider the needs of different levels of staff, organizational access and using capital/cost effectiveness and organizing corrective action, strategic thinking in organizing personnel (14), related skills and knowledge necessary to access, adapt and apply EBM (13), understanding the role of the main stakeholders in health and treatment and how they work, understanding how specialized health and treatment forces work, creating a special accountability structure for the health and treatment structure and effectively identifying organizational structures, roles and communications to achieve the organization’s goals, interpreting basic financial conditions, determining budgets based on Organizational goals, designing and creating appropriate roles and describing the structure in line with organizational goals and creating mutual performance reviews (16)
2	Leadership (3, 14–17)	Role modeling, motivating others, sharing information and experiences and effective transfer of information (3), adaptability, self-confidence, empowerment and commitment to empowerment and safe change (17), leadership and management of others, planning and Implementation of change, honest self-evaluation ability and professional and managerial ethics (15), leadership of change, organizational awareness, strategic adaptation, social adaptation, evaluation of organizational characteristics that lead to fluctuation in employees (14), balance of values and priorities Organizational and professional, leadership, guidance and performance evaluation to build an effective team, adapting the leadership style to suit the conditions and creating and maintaining a professional and individual support network, defining the need for change in an effective way, assessing readiness for change and implementation It, process evaluation and change output (16)
3	Personal characteristics and attitude (3, 13, 14, 16, 17)	Adapting to change and adapting to priorities, openness to stakeholders’ data and openness to a person’s change of mind (3), personal style, attitude, and essential characteristics to apply EBM (13, 17), and creative thinking., work interdependence (14), recognizing and tolerating ambiguity (16)
4	Communication (3, 13–17)	Emotional intelligence, conflict management skills, relationship building skills, open door policy, accepting the mistakes of others (3), essential abilities to communicate with others, communication skills to communicate with peers and stakeholders, negotiation skills to ensure partnership and commitment from colleagues. And other professions, effective knowledge to motivate others and the ability to work closely with other professions (17), interpersonal communication, writing ability, speaking with groups, presentation skills and working in teams (15), Establishing constructive communication between new departments (14), the necessary ability to communicate and act effectively with others (13), participating confidently and constructively in verbal and non-verbal interactions with others, preparing a report that is suitable for both the audience and the purpose. Spending time and effort to work and interact with the stakeholders and show awareness and sensitivity towards the feelings of others (16)
5	Information management (13, 14, 17)	Ability to obtain, manage, and optimal use of information, awareness, and evaluation of resources including economic profit and loss (17), information technology management (14), management and effective use of information (13)
6	Self-management (3, 13, 17)	Self-empowerment and process and quality improvement (3), time management, effective organization and responsibility for personal tasks in the workplace (17), organization and responsibility for individual actions in the workplace (13)
7	Way of thinking (3, 14, 16)	Systemic thinking, comprehensive thinking, long-term thinking, analytical thinking, systematic thinking, innovation and resourcefulness (3), development of talents (14), determining and using criteria to evaluate the results of decisions, and supporting and encouraging colleagues and subordinates to use evidence. to make a decision (16)
8	Research and use of evidence (3, 13, 16, 17)	Ethics in research, knowledge in searching and understanding data, knowledge in data collection, knowledge in data analysis and practical application (3), statistical knowledge to calculate the risk related to research findings and their conceptualization on the patient (17), including Timely and appropriate use of question/evaluation to determine the nature of the problem, issue or opportunity, use of evidence to question and improve current practices and processes, determine and use criteria to evaluate the results of decisions (16), identify valid research, evaluate Criticizing the validity of research evidence and judging the validity of research findings and arguments (13)

## Discussion

The purpose of this research was to use a domain review approach to explain the evidence-based competency requirements of health managers. The findings of this research show that to examine evidence-based competencies in managers, attention should be paid to the following: professional and technical knowledge, leadership, personal characteristics and attitude, communication, information management, self-management, way of thinking, and research and use of the evidence.

### Professional and technical knowledge

Through this study, the first competency that was acquired was professional and technical knowledge. This encompasses the essential knowledge and skills required to effectively manage organizational activities, including general management, evidence-based protocols, corrective actions, problem-solving, mastery of organizational processes, and quality assurance. Jahormi notes that skepticism towards knowledge has always been present, and scientific ignorance remains a significant barrier to the acceptance of EBM among managers (18). Similarly, Zare et al. highlight that the lack of skills and expertise related to scientific evidence is an obstacle to taking action based on evidence (19). Despite the use of numerous educational strategies, Albarconi et al. note that the lack of knowledge and skills related to evidence-based practices remains one of the most frequently reported barriers to implementation (20). Experts in evidence-based practice emphasize the importance of nurses being attuned to the continually evolving scientific knowledge and possessing the critical judgment necessary to conduct evidence-based practices (21).

Effective management is crucial for the successful planning, implementation, and monitoring of organizational activities, while financial management allows for the budgeting and planning of these activities (11). Process management involves the use of various methods, tools, and techniques to enhance the quality of healthcare service provision (22). Additionally, human resources, through the implementation of activities and strategies, ensure the successful management of employees, aligning them towards the organization’s objectives (23). Understanding the beneficiaries is also vital as it allows stakeholders to provide different medical services with varying resource inputs and behavioral participation, resulting in different service effects. Therefore, organizational units must explore their intertwined position and values to achieve joint value creation (24). To be an evidence-based manager, it is essential to possess the skills and knowledge required to access, adapt, and apply EBM. However, the decision-making process should not be limited to the “best available evidence” as other factors may come into play. Therefore, it is necessary to consider evidence that best matches the context of the organization (2). Technical professional knowledge, which is heavily reliant on management-related education, plays an essential role in obtaining and implementing evidence in healthcare organizations.

### Leadership

As per our study, the ability to lead was identified as a crucial competency. A strong leader is essential in enhancing productivity and organizational capabilities. Effective leadership plays a pivotal role in bringing about the required changes to elevate the quality of organizations. This domain encompasses skills such as guiding others, driving change, fostering a conducive environment for individuals, facilitating smooth communication, and empowering team members. According to the research conducted by Steller et al., leaders who strategically implemented evidence-based elements observed a positive effect on the behavior of their managers and employees over time (25). Omar in his study. Regarding the role of the leader in the implementation of evidence-based practice, he says that the initial decision to start an implementation effort in the hospital and subsequent continuous changes during the implementation process requires the participation of leadership at different levels and from multiple stakeholders in the hospital departments (26). Healthcare leaders who are more spiritually developed can achieve significantly more positive results for their organization by challenging the process, sharing vision, and motivating others to work in a classical way (27). Quality improvement requires change, and effective leadership is the key to change. The specific structure of healthcare organizations can act as a barrier to change (28). Role modeling by adopting practices for example and encouraging acceptance in others is another manifestation of leadership ability (3). The findings of Monica Bianchi et al.’s study showed that managers have a particularly influential role in the implementation of EBM in terms of providing a supportive culture and environment. For this, they must have basic knowledge, but also be aware of and address implementation barriers, and understand the key role of managers in creating and supporting the optimal environment (29). Shelley et al. about the important role of leaders in the implementation of evidence in the organization believe that leaders try to optimize the success of the implementation by expressing passion for change in the form of presence, support, and attention to the implementation process; and showing willingness to receive feedback from employees regarding change. Leadership that lacks authority and does not support change, or neglects to hold employees accountable for change, creates barriers to implementation (30). Empowerment, influence, and negotiation skills, to obtain the commitment of others (including the group peers, nurses, and therapists) and successful change depend on teamwork at different levels and health care professionals (31). Leadership as one of the key skills requires environmental awareness and organizational knowledge in addition to extensive knowledge. Having this skill seems to be very useful for making evidence-based changes and promoting the use of evidence in the organization.

### Personal characteristics and attitude

Our study identified the third competency as personal characteristics and attitude. We found that the necessary attitude and characteristics to apply EBM, creative thinking, and openness to changes in people’s opinions were crucial in this field. According to Kremer and colleagues’ research, knowledge sharing is a key success factor that leads to creativity and innovation. Employees benefit greatly from the knowledge accumulated in the organization through means such as knowledge sharing. Furthermore, accumulated knowledge helps foster creativity and innovation, and includes elements such as organizational culture and identity, policies, routines, systems, and other employees (32). According to a study conducted by Cleven et al., implementing process innovation in hospitals can lead to increased efficiency and improved quality of care, ultimately resulting in reduced workload and increased job satisfaction for healthcare professionals (33). It is crucial to remain open to changing ideas and decisions, especially when new evidence suggests a different direction (3). In a study by Abelson et al., they found that managers often struggled with the tolerance of ambiguity, particularly when it came to balancing personal beliefs about patient care with the organizational need to produce positive health outcomes (34). Research shows that competence is an essential and enduring aspect of one’s personality, with the ability to forecast behavior and performance across various situations and professional responsibilities. This indicates that competency serves as a precursor to behavior and performance, helping to identify individuals who excel or struggle in a given task (13). As such, a manager’s traits and outlook are fundamental in gathering and applying evidence. Given their role as primary decision-makers, managers must cultivate this skill to leverage evidence effectively and adapt their decisions as needed.

### Communication

Effective communication is a highly valued attribute that was frequently emphasized in the studies we examined. Communication proficiency, conflict resolution, negotiation tactics, and team collaboration were all considered essential in this category. According to Hasanpoor’s research on the obstacles to implementing EBM, inadequate communication between knowledge producers and hospital decision-makers was deemed the most significant hindrance. Knowledge producers are those who generate research-based, practice-based, or experience-based knowledge that can be disseminated to hospital decision-makers, such as nursing managers (35). According to the implementation of evidence-based practice, effective communication among healthcare professionals in a Dutch ICU was a crucial obstacle in launching a tiered scoring system successfully (36). Diedrick adds that devising a well-planned communication strategy for implementing EBP changes, which is formulated with staff’s input and grounded in evidence, can enhance nurse contentment (37). Powell’s research demonstrated that the combination of a cohesive team and a strong belief in evidence-based practices resulted in a high adoption rate within clinics. Conversely, when evidence was disregarded, teamwork proved valuable in bolstering resistance to enforcement efforts. In particular, effective teamwork was critical in new programs requiring the involvement of specialists within multispecialty teams (38). The human relations skill, which encompasses the ability to cultivate an environment of coordination and collaboration, delegate tasks, function effectively as a team member, comprehend people’s motivations, and influence their behavior, was found to be highly relevant (14). To succeed in organizations and be an effective manager, having strong interpersonal skills is crucial (39). Relationship management involves the ability to initiate, foster, and maintain positive relationships with colleagues. It also includes the ability to establish professional connections with individuals both within and outside the organization, which can facilitate the sharing of information and expert perspectives (3). Employees’ interpersonal skills are closely linked to their attitudes and performance at both the individual and team levels, and ultimately impact the productivity of the organization as well as long-term job performance in healthcare settings (40). Given that teamwork and interpersonal communication are integral to organizational success, managers must prioritize the development of these skills to implement evidence-based practices within the organization.

### Information management

The ability to effectively manage information is a crucial skill known as information management. It encompasses managing information, being aware of and evaluating available resources, and utilizing information correctly. Healthcare workers, including policymakers, hospital managers, and health professionals like doctors, nurses, and therapists, make diverse decisions and judgments daily based on a range of evidence, from strong scientific evidence to peer opinions and local data sources, while also considering patients’ preferences. Depending on the situation, different data and levels of evidence are required. Misuse of these sources can lead to suboptimal decisions and outcomes, as noted in reference (41). Filtering information, organizing new information in a manageable form, keeping it up-to-date, and being in the course of current developments in the field of work are the characteristics of this dimension (13). According to Perez, with technological advances in the field of information technology, most managers will no longer try to collect information, but instead, so much information will be available that selecting, analyzing, and understanding the available evidence will become increasingly important. Managers can facilitate the implementation of EBM in their organization by investing in the capabilities of their information systems to enable efficient data collection and storage and easy access throughout the organization (42). The practice of information technology management involves ensuring that information needs and strategies are in sync with the development and maintenance of technology. One important aspect of this is enhancing the usefulness of technology by ensuring compatibility with external links such as health service users, suppliers, and other providers within the healthcare industry (14). As the use of information technology continues to evolve and generate vast amounts of data across different levels of healthcare organizations, it becomes increasingly important for evidence-based managers to possess competencies in utilizing information accurately and converting data into evidence.

### Self-management

The study’s next competency is self-management, encompassing self-reliance, time management, improvement, and accountability. Healthcare managers are responsible for both patients and their organizations, and an evidence-based manager is empowered to effect change (43). Self-improvement is a critical component, involving the development of personal skills, learning from missteps, and taking the initiative to learn and grow (3). Effective management involves self-reflection and aligning personal goals with the requirements of the organization, even in the face of necessary changes that may meet resistance from some individuals (44). This applies to both general management and environmental management. For instance, it is crucial to develop a comprehensive understanding of the interplay between social, economic, demographic, and environmental trends, and to conduct voluntary research regarding the impact of organizational processes, products, or services on the work environment (45). Effective time management skills are a crucial component in the workplace, as they can significantly impact both personal and organizational outcomes. Research has demonstrated that the use of time management skills can enhance job performance, work prioritization, project acceleration, and overall job satisfaction (46). Additionally, studies have found that many nurses tend to rely on habitual or traditional nursing practices, which may be linked to inadequate time management skills (47). As managers are instrumental in the implementation of evidence-based practices, developing self-management competency is a critical step for managers to become more effective in implementing evidence within their organizations.

### Way of thinking

Our study revealed that thinking skills were a significant factor in achieving success. These skills included comprehensive, systemic, creative, analytical, and long-term thinking. Avery’s research highlights the importance of comprehensive and long-term thinking in recognizing the value of building and maintaining relationships with key stakeholders (3). In the healthcare industry, which is characterized by high levels of dynamic complexity, dynamic systems thinking has proven effective in analyzing and designing policies. This approach has been applied to areas such as infectious disease transmission, screening program effectiveness, primary care systems design, and waiting list escalation causation (48). As demonstrated by Uloaku’s research, a clear correlation exists between implementing a systemic approach and successfully reducing patient wait times (49). Additionally, an evidence-based manager must possess the skills necessary to assess decision outcomes and foster a culture of evidence-based decision-making among their colleagues and subordinates (22). By adopting this mindset, managers can gain valuable insights into their organization, analyze its operations, and drive innovation. The benefits of equipping managers with these abilities, and the ability to apply evidence effectively, are undeniable.

### Research and use of evidence

The final competency uncovered by this study pertained to research and the utilization of evidence. The studies revealed that this involves expertise in gathering and analyzing data, as well as a strong grounding in ethical research practices. An understanding of data collection procedures, from recording to accuracy assessment, as well as familiarity with various statistical analysis methods, all fall under the umbrella of research knowledge (3). To conduct effective research, one must be able to identify key terms, extract pertinent information, and navigate relevant databases. Familiarity with search terms is also a crucial prerequisite for successful research (15). Raines and Bartunek emphasized the crucial role of utilizing appropriate theories, interpretive frameworks, routines, cues, and learning in converting a vast amount of research data into accessible and high-quality information (50). McCormick further stressed that ethical analysis must be the core of any EBM decision-making process and should be addressed early on or at the beginning of the process (51). Additionally, honesty about the data used in decision-making is a fundamental aspect that must be upheld (3). The utilization of evidence to scrutinize and enhance existing practices and procedures, along with ascertaining and implementing standards to appraise the outcomes of choices, are labeled as the key elements of evidence employment in this dimension (14). Research-derived data is deemed as one of the most crucial forms of evidence for managers. Equipping themselves with the skill to research and access the latest and reliable evidence will enable managers to make more informed decisions.

## Conclusion

This article delves into the various factors that contribute to the competency of evidence-based managers in healthcare. Given the relatively recent emergence of EBM science, there is limited literature on the subject, particularly when it comes to assessing competence. Upon examining published articles in the field, it becomes clear that previous studies primarily focused on the ability to access information and use evidence, while recent studies have shifted towards more abstract skills such as communication, self-management, and critical thinking. With ongoing changes in healthcare, including the advent of new technologies and the production of vast amounts of data, evidence-based managers must continually develop their abilities to access and obtain evidence, as well as hone their interpersonal and intra-personal skills. According to the article’s authors, additional applied studies are needed to further enhance our understanding of evidence-based manager competency in the context of healthcare and treatment.

## Data Availability

The original contributions presented in the study are included in the article/Supplementary material, further inquiries can be directed to the corresponding author.

## References

[1] Provost F Fawcett T. Data science for business: What you need to know about data mining and data-analytic thinking. 1st edn. O'Reilly Media, Inc. (2013).

[2] Starkey K Hatchuel A Tempest S. Management research and the new logics of discovery and engagement. J Manag Stud. (2009) 46:547–58. doi: 10.1111/j.1467-6486.2009.00833.x

[3] Daouk-Öyry L Sahakian T van de Vijver F. Evidence-based management competency model for managers in hospital settings. Br J Manag. (2021) 32:1384–403. doi: 10.1111/1467-8551.12434

[4] Sahakian T. Evidence-based management in hospital settings: Unraveling the process and the role of the person and the context. Design Kesdenian. (2020).

[5] Kovner AR Rundall TG. Evidence-based management reconsidered. Front Health Serv Manag. (2006) 22:3–22. doi: 10.1097/01974520-200601000-00002 16604900

[6] Wright AL Zammuto RF Liesch PW Middleton S Hibbert P Burke J . Evidence-based management in practice: opening up the decision process, decision-maker and context. Br J Manag. (2016) 27:161–78. doi: 10.1111/1467-8551.12123

[7] Yang K. What can COVID-19 tell us about evidence-based management? Am Rev Public Adm. (2020) 50:706–12. doi: 10.1177/0275074020942406

[8] Alexander JA Hearld LR Jiang HJ Fraser I. Increasing the relevance of research to health care managers: hospital CEO imperatives for improving quality and lowering costs. Health Care Manag Rev. (2007) 32:150–9. doi: 10.1097/01.HMR.0000267792.09686.e3, PMID: 17438398

[9] Kovner AR Elton J Billings J. Transforming health management: an evidence-based approach. Front Health Serv Manag. (2000) 16:3–24. doi: 10.1097/01974520-200004000-00002 11183283

[10] Pfeffer J Sutton RI. Evidence-based management. Harv Bus Rev. (2006) 84:62–74, 133. PMID: 16447370

[11] Arksey H O'Malley L. Scoping studies: towards a methodological framework. Int J Soc Res Methodol. (2005) 8:19–32. doi: 10.1080/1364557032000119616

[12] Tricco AC Lillie E Zarin W O'Brien KK Colquhoun H Levac D . PRISMA extension for scoping reviews (PRISMA-ScR): checklist and explanation. Ann Intern Med. (2018) 169:467–73. doi: 10.7326/M18-0850, PMID: 30178033

[13] Cowling A Newman K Leigh S. Developing a competency framework to support training in evidence-based healthcare. Int J Health Care Qual Assur. (1999) 12:149–60. doi: 10.1108/09526869910272509

[14] Liang Z Blackstock FC Howard PF Briggs DS Leggat SG Wollersheim D . An evidence-based approach to understanding the competency development needs of the health service management workforce in Australia. BMC Health Serv Res. (2018) 18:1–12. doi: 10.1186/s12913-018-3760-z, PMID: 30563505 PMC6299513

[15] Newman K Pyne T Leigh S Rounce K Cowling A. Personal and organizational competencies requisite for the adoption and implementation of evidence-based healthcare. Health Serv Manag Res. (2000) 13:97–110. doi: 10.1177/095148480001300204, PMID: 11184014

[16] White KR Clement DG Nayar P. Management competency evaluation: alumni perceptions. J Health Adm Educ. (2006) 23:335–49. 17503702

[17] Martins J Isouard G. An evidence-based framework: competencies and skills for managers in Australian health services. Asia Pacific J Health Manag. (2015) 10:8–23.

[18] Pazhohesh-Jahromy A. Evidence-based management: A bridge for the gap between the management science and manager practice. Technol. Dev. (Roshd-e-Fanavari) (2017) 13:52–61.

[19] Zare H Khanifar H Yazdani HR Ahmadi Azarm H. Evidence-based human resource management: Systematic review and qualitative interpretative meta-synthesis (QIMS). Organ. Res. Methods. (2019) 9:115–40.

[20] Albarqouni L Hoffmann T Straus S Olsen NR Young T Ilic D . Core competencies in evidence-based practice for health professionals: consensus statement based on a systematic review and Delphi survey. JAMA Netw Open. (2018) 1:1–12. doi: 10.1001/jamanetworkopen.2018.0281 30646073

[21] Yoo JY Kim JH Kim JS Kim HL Ki JS. Clinical nurses’ beliefs, knowledge, organizational readiness and level of implementation of evidence-based practice: the first step to creating an evidence-based practice culture. PLoS One. (2019) 14:e0226742. doi: 10.1371/journal.pone.0226742, PMID: 31877147 PMC6932768

[22] Taylor MJ McNicholas C Nicolay C Darzi A Bell D Reed JE. Systematic review of the application of the plan–do–study–act method to improve quality in healthcare. BMJ Qual Safety. (2013) 23:290–8. doi: 10.1136/bmjqs-2013-001862, PMID: 24025320 PMC3963536

[23] Elarabi HM Johari F. The impact of human resources management on healthcare quality. Asian J Manag Sci Educ. (2014) 3:13–22.

[24] Wu J Wang Y Tao L Peng J. Stakeholders in the healthcare service ecosystem. Procedia CIRP. (2019) 83:375–9. doi: 10.1016/j.procir.2019.04.085

[25] Stetler CB Ritchie JA Rycroft-Malone J Charns MP. Leadership for evidence-based practice: strategic and functional behaviors for institutionalizing EBP. Worldviews Evid-Based Nurs. (2014) 11:219–26. doi: 10.1111/wvn.12044, PMID: 24986669 PMC4240461

[26] Omer T. Research utilization in a multicultural nursing setting in Saudi Arabia: barriers and facilitators. J Nurs Res. (2012) 20:66–73. doi: 10.1097/JNR.0b013e31824777d8, PMID: 22333967

[27] Chatterjee R Suy R Yen Y Chhay L. Literature review on leadership in healthcare management. J Soc Sci Stud. (2018) 5:38–47. doi: 10.5296/jsss.v5i1.11460, PMID: 39775451

[28] Kumar RD. Leadership in healthcare. Anaesth Intens Care Med. (2013) 14:39–41. doi: 10.1016/j.mpaic.2012.11.006

[29] Bianchi M Bagnasco A Bressan V Barisone M Timmins F Rossi S . A review of the role of nurse leadership in promoting and sustaining evidence-based practice. J Nurs Manag. (2018) 26:918–32. doi: 10.1111/jonm.12638, PMID: 30198088

[30] Li S-A Jeffs L Barwick M Stevens B. Organizational contextual features that influence the implementation of evidence-based practices across healthcare settings: a systematic integrative review. Syst Rev. (2018) 7:1–19. doi: 10.1186/s13643-018-0734-5 29729669 PMC5936626

[31] Stanley D. Multigenerational workforce issues and their implications for leadership in nursing. J Nurs Manag. (2010) 18:846–52. doi: 10.1111/j.1365-2834.2010.01158.x, PMID: 20946220

[32] Kremer H Villamor I Aguinis H. Innovation leadership: best-practice recommendations for promoting employee creativity, voice, and knowledge sharing. Bus Horiz. (2019) 62:65–74. doi: 10.1016/j.bushor.2018.08.010

[33] Cleven A Mettler T Rohner P Winter R. Healthcare quality innovation and performance through process orientation: evidence from general hospitals in Switzerland. Technol Forecast Soc Chang. (2016) 113:386–95. doi: 10.1016/j.techfore.2016.07.007

[34] Abelsson T Karlsson A-K Morténius H. A feeling of ambiguity: a qualitative content analysis of managers’ experiences of evidence-based practice in Swedish primary care. Journal of healthcare. Leadership. (2022) 14:143–53. doi: 10.2147/JHL.S371643, PMID: 36160473 PMC9507276

[35] Hasanpoor E Siraneh Belete Y Janati A Hajebrahimi S Haghgoshayie E. Nursing managers’ perspectives on the facilitators and barriers to implementation of evidence-based management. Worldviews Evid-Based Nurs. (2019) 16:255–62. doi: 10.1111/wvn.12372, PMID: 31155846

[36] Riekerk B Pen EJ Hofhuis JG Rommes JH Schultz MJ Spronk PE. Limitations and practicalities of CAM-ICU implementation, a delirium scoring system, in a Dutch intensive care unit. Intens Critic Care Nurs. (2009) 25:242–9. doi: 10.1016/j.iccn.2009.04.001, PMID: 19540761

[37] Diedrick LA Schaffer MA Sandau KE. A practical communication strategy to improve implementation of evidence-based practice. JONA. J Nurs Adm. (2011) 41:459–65. doi: 10.1097/NNA.0b013e3182346e61 22033315

[38] Powell A Davies H Bannister J Macrae W. Understanding the challenges of service change–learning from acute pain services in the UK. J R Soc Med. (2009) 102:62–8. doi: 10.1258/jrsm.2008.080194, PMID: 19208870 PMC2642864

[39] Beenen G Pichler S Davoudpour S. Interpersonal skills in MBA admissions: how are they conceptualized and assessed? J Manag Educ. (2018) 42:34–54. doi: 10.1177/1052562917703743, PMID: 39777019

[40] Lievens F Sackett PR. The validity of interpersonal skills assessment via situational judgment tests for predicting academic success and job performance. J Appl Psychol. (2012) 97:460–8. doi: 10.1037/a0025741, PMID: 21967295

[41] Roshanghalb A Lettieri E Aloini D Cannavacciuolo L Gitto S Visintin F. What evidence on evidence-based management in healthcare? Manag Decis. (2018) 56:2069–84. doi: 10.1108/MD-10-2017-1022

[42] Criado-Perez C. Measuring and predicting the use of evidence-based management UNSW Sydney (2021).

[43] Guo R Berkshire SD Fulton LV Hermanson PM. Use of evidence-based management in healthcare administration decision-making. Leadersh Health Serv. (2017) 30:330–42. doi: 10.1108/LHS-07-2016-0033, PMID: 28693398

[44] McConnell CR. Self-management: key to success as a manager. Health Care Manag. (2010) 29:83–93. doi: 10.1097/HCM.0b013e3181cd8c4d, PMID: 20145473

[45] Ciocirlan CE. Environmental workplace behaviors: definition matters. Organ Environ. (2017) 30:51–70. doi: 10.1177/1086026615628036

[46] Bahadori M Salesi M Ravangard R Hosseini SM Raadabadi M Hojati Dana A . Prioritization of factors affecting time management among health managers. Int J Travel Med Glob Health. (2015) 3:159–64. doi: 10.20286/ijtmgh-3360304142

[47] Rahmayanti EI Kadar KS Saleh A. Readiness, barriers and potential strenght of nursing in implementing evidence-based practice. Int J Caring Sci. (2020) 13:1203–11.

[48] Lebcir M. Health care management: the contribution of systems thinking. (2006).

[49] Uloaku OL Grace MO. Strategic thinking and patient waiting time in teaching hospitals in Ogun state, Nigeria. (2021) 8:71–7.

[50] Rynes SL Bartunek JM. Evidence-based management: foundations, development, controversies and future. Annu Rev Organ Psych Organ Behav. (2017) 4:235–61. doi: 10.1146/annurev-orgpsych-032516-113306

[51] McCormick DW. Ethics & the 15 minute evidence-based manager: a review of a response to a critique published as “evidence-based management: concept cleanup time?” by rob B briner, David Denyer, and Denise M Rousseau (academy of management perspectives, Nov 2009). Org Manag J. (2010) 7:303–6. doi: 10.1057/omj.2010.40

