# Supplementary material for: Requirements for evidence-based management competency in healthcare: a scoping review
Source: Front Public Health. 2025 Jan 28;13:1490454. doi: 10.3389/fpubh.2025.1490454 (PMC11810947; doi:10.3389/fpubh.2025.1490454)
Supplement: Supplementary file 1 [file Table_1.DOCX]

("evidence based management" OR “Evidence Based Health Care Management” OR “Evidence Based Healthcare Management” OR “health care managers” OR “health care management” OR “health service manager” OR “health service managers” OR “health service management” OR “management competency” OR “management competencies” OR “Clinical Competence” OR “Clinical Competency” OR “Clinical Competencies” OR “Clinical Skill” OR “Clinical Skills” OR “Professional Competence” OR “Generalization of Expertise” OR “Expertise Generalization” OR “Technical Expertise” OR “Evidence-Based Practice” OR “Evidence-Based Health Care” OR “Evidence-Based Healthcare” OR “Evidence-Based Healthcares” OR “Evidence Based Health Care Management”) AND (hospitals OR hospital OR “health care organization”)

| Search String | Database | Results |
| --- | --- | --- |
| ("evidence based management"[TIAB] OR “Evidence Based Health Care Management”[TIAB] OR “Evidence Based Healthcare Management”[TIAB] OR “health care managers”[TIAB] OR “health care management”[TIAB] OR “health service manager”[TIAB] OR “health service managers”[TIAB] OR “health service management”[TIAB] OR “management competency”[TIAB] OR “management competencies”[TIAB] OR “Clinical Competence”[MH] OR “Clinical Competence”[TIAB] OR “Clinical Competency”[TIAB] OR “Clinical Competencies”[TIAB] OR “Clinical Skill*”[TIAB] OR “Professional Competence”[MH] OR “Professional Competence”[TIAB] OR “Generalization of Expertise”[TIAB] OR “Expertise Generalization”[TIAB] OR “Technical Expertise”[TIAB] OR “Evidence-Based Practice”[MH] OR “Evidence-Based Practice”[TIAB] OR “Evidence-Based Health Care”[TIAB] OR “Evidence-Based Healthcare”[TIAB] OR “Evidence-Based Healthcares”[TIAB] OR “Evidence Based Health Care Management”[TIAB]) AND (hospitals[MH] OR hospitals[TIAB] OR hospital[TIAB] OR “health care organization”[TIAB]) | PubMed | 24725 |
| TS=("evidence based management" OR “Evidence Based Health Care Management” OR “Evidence Based Healthcare Management” OR “health care managers” OR “health care management” OR “health service manager” OR “health service managers” OR “health service management” OR “management competency” OR “management competencies” OR “Clinical Competence” OR “Clinical Competency” OR “Clinical Competencies” OR “Clinical Skill” OR “Clinical Skills” OR “Professional Competence” OR “Generalization of Expertise” OR “Expertise Generalization” OR “Technical Expertise” OR “Evidence-Based Practice” OR “Evidence-Based Health Care” OR “Evidence-Based Healthcare” OR “Evidence-Based Healthcares” OR “Evidence Based Health Care Management”) AND TS=(hospitals OR hospital OR “health care organization”) | WOS | 5338 |
| TITLE-ABS-KEY("evidence based management" OR “Evidence Based Health Care Management” OR “Evidence Based Healthcare Management” OR “health care managers” OR “health care management” OR “health service manager” OR “health service managers” OR “health service management” OR “management competency” OR “management competencies” OR “Clinical Competence” OR “Clinical Competency” OR “Clinical Competencies” OR “Clinical Skill” OR “Clinical Skills” OR “Professional Competence” OR “Generalization of Expertise” OR “Expertise Generalization” OR “Technical Expertise” OR “Evidence-Based Practice” OR “Evidence-Based Health Care” OR “Evidence-Based Healthcare” OR “Evidence-Based Healthcares” OR “Evidence Based Health Care Management”) AND TITLE-ABS-KEY (hospitals OR hospital OR “health care organization”) | Scopus | 56509 |

7 Oct 2023
